# Non-rigid image registration of 4D-MRI data for improved delineation of moving tumors

**DOI:** 10.1186/s12880-020-00439-6

**Published:** 2020-04-23

**Authors:** Stefan Weick, Kathrin Breuer, Anne Richter, Florian Exner, Serge-Peer Ströhle, Paul Lutyj, Jörg Tamihardja, Simon Veldhoen, Michael Flentje, Bülent Polat

**Affiliations:** 1 Department of Radiation Oncology, University of Wuerzburg, Josef-Schneider-Str. 11, 97080 Wuerzburg, Germany; 2 Department of Diagnostic and Interventional Radiology, University of Wuerzburg, Wuerzburg, Germany

**Keywords:** 4D-MRI, Respiratory induced tumor motion, Non-rigid image registration, Radiotherapy treatment planning

## Abstract

**Background:**

To increase the image quality of end-expiratory and end-inspiratory phases of retrospective respiratory self-gated 4D MRI data sets using non-rigid image registration for improved target delineation of moving tumors.

**Methods:**

End-expiratory and end-inspiratory phases of volunteer and patient 4D MRI data sets are used as targets for non-rigid image registration of all other phases using two different registration schemes: In the first, all phases are registered directly (dir-Reg) while next neighbors are successively registered until the target is reached in the second (nn-Reg). Resulting data sets are quantitatively compared using diaphragm and tumor sharpness and the coefficient of variation of regions of interest in the lung, liver, and heart. Qualitative assessment of the patient data regarding noise level, tumor delineation, and overall image quality was performed by blinded reading based on a 4 point Likert scale.

**Results:**

The median coefficient of variation was lower for both registration schemes compared to the target. Median dir-Reg coefficient of variation of all ROIs was 5.6% lower for expiration and 7.0% lower for inspiration compared with nn-Reg. Statistical significant differences between the two schemes were found in all comparisons. Median sharpness in inspiration is lower compared to expiration sharpness in all cases. Registered data sets were rated better compared to the targets in all categories. Over all categories, mean expiration scores were 2.92 ± 0.18 for the target, 3.19 ± 0.22 for nn-Reg and 3.56 ± 0.14 for dir-Reg and mean inspiration scores 2.25 ± 0.12 for the target, 2.72 ± 215 0.04 for nn-Reg and 3.78 ± 0.04 for dir-Reg.

**Conclusions:**

In this work, end-expiratory and inspiratory phases of a 4D MRI data sets are used as targets for non-rigid image registration of all other phases. It is qualitatively and quantitatively shown that image quality of the targets can be significantly enhanced leading to improved target delineation of moving tumors.

## Background

Precise information regarding the extent of respiratory induced motion of tumors and surrounding organs at risk (OAR) plays a crucial role in radiotherapy treatment (RT) and planning. Highly conformal treatment techniques require an exact target and OAR delineation in treatment planning. Treatment planning concepts using the mid-ventilation and internal-target volume concept [1, 2] are based on the extent of tumor motion between expiration and inspiration. Four-dimensional (4D) imaging is therefore required to provide necessary information about the individual respiration-associated motion patterns [3, 4]. In this context, 4D computed tomography (CT) is commonly used as the basis for treatment planning [5]. External [6] or internal [7, 8] respiratory surrogates can be acquired simultaneously during free-breathing CT imaging to retrospectively sort the acquired data into different respiratory phases (bins) and hence to avoid motion artifacts [9, 10]. Magnetic resonance imaging (MRI) is an alternative method to acquire 4D data sets. MRI is characterized by its superior soft-tissue contrast which is important for the identification of lesions e.g. within the prostate, the liver or the pancreas [11–13].

Free-breathing MRI, where the MR data itself serves as an internal respiratory surrogate (‘self-gating’), can be used to retrospectively reconstruct 4D data sets with reduced motion artifacts. For this, there are no additional devices compared to 4D CT necessary. Radial [14, 15] and Cartesian [16–18] acquisition techniques using ‘self-gating’ were proposed. These techniques commonly make use of compressed sensing or parallel imaging reconstructions to compensate inherently long acquisition times due to the large number of phase encoding steps, which have to be acquired in 4D MRI. This allows for clinically acceptable examination times and image quality in terms of achievable resolution and signal to noise ratio (SNR).

The acquired respiratory signal is divided into several gating windows (bins) which represent different respiratory phases. Usually, 10 different respiratory phases from inspiration to expiration are reconstructed [19]. Small bins are characterized by good motion artifact reduction (blurring and ghosting) but also reduced SNR or even under-sampling artifacts due to missing lines after gating [20, 21]. In contrast, larger bins lead to increased SNR but come along with increased motion artifacts. Artifacts resulting from motion and under-sampling as wells as low SNR degrade the diagnostic value of the 4D MRI data set. This is particularly important in target volume definition, which is often based on the identification of the maximal tumor displacement between end-inspiration and end-expiration. Therefore, sufficient motion compensation and at the same time high SNR have to be achieved in these phases to allow for high delineation accuracy.

In this work, end-expiration and end-inspiration are used as target volumes for non-rigid image registration for the reconstruction of 4D data sets acquired under free-breathing conditions using respiratory self-gating. The resulting data sets are quantitatively and qualitatively compared to investigate and compare the diagnostic value of both registration schemes.

## Methods

### Data acquisition and reconstruction

**Fig. 1:**
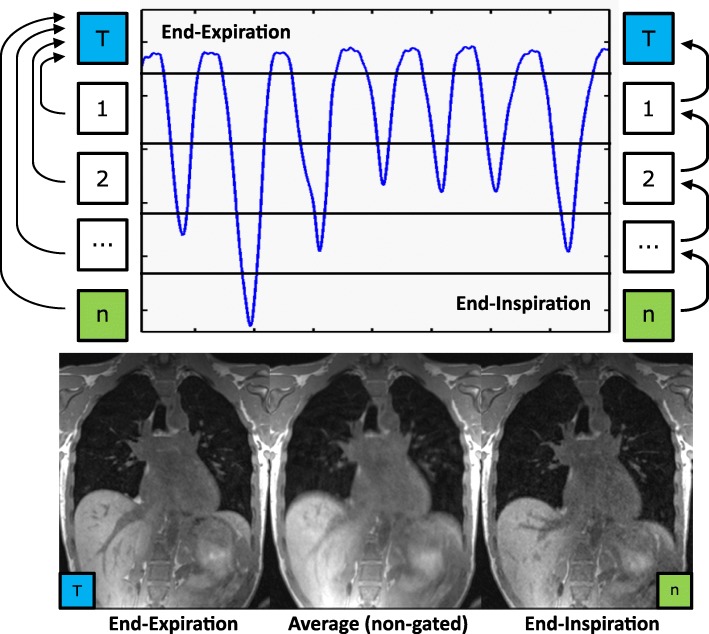
Schematic illustration of the two registration schemes with the end-expiratory phase as target (T) and time course of the respiratory signal with gating bins. Left: All phases are directly registered onto T. Right: Starting with inspiration, adjacent phases are subsequently registered until T is reached. End-expiration, end-inspiration and a non-gated image are displayed at the bottom to emphasize differences in signal intensity, sharpness and motion artifacts

A SIEMENS 1.5 Tesla scanner (Magnetom Avanto, Siemens Healthineers, Erlangen, Germany) was used for all experiments. A six-channel body array in combination with a spine array was used for signal reception. Data were acquired under free-breathing conditions with a recently proposed 3D Cartesian FLASH sequence, characterized by a non-uniform order of phase encoding steps [16]. The central k-space signal was used as a navigator signal for retrospective respiratory self-gating [21]. Data were sorted into ten different gating windows (bins), representing different respiratory phases from end-expiration to end-inspiration, using equal bin intervals. A 16-core Intel Xeon CPU system with 125 GB RAM and MATLAB (Math Works, Natick, Massachusetts) was used for data reconstruction and registration. Missing k-space lines after the gating process were reconstructed using an iterative parallel imaging algorithm (SPIRiT) [22]. An exemplary time course of the respiratory signal divided into different gating bins of a volunteer measurement is shown at the top of Fig. 1. A slice of the corresponding expiratory, inspiratory and averaged free-breathing data set is shown at the bottom. The averaged data set is characterized by high signal intensity (see liver) but distinctive motion artifacts (see diaphragm) whereas the gated data set shows strongly reduced blurring but also decreased signal intensity.

The following imaging parameters were used: echo time TE = 1.2 ms, repetition time TR = 4.0 ms, flip angle = 10°, Field of View FoV = 400 × 400 × 185 mm^3^, bandwidth = 1002 Hz/px, coronal orientation, matrix size = 192 × 192 × 88, resulting isotropic resolution = 2.1 mm^3^. The high receiver bandwidth was used to mitigate distortions caused by chemical shift and magnetic susceptibility differences. The number of phase encoding steps was adjusted to a total acquisition time of 180 s except for one patient measurement to 300 s. Six healthy volunteers (3 male, 3 female, age 31–60 yrs) and four patients (2 male, 2 female, age 65–70 yrs), with liver and adrenal gland metastasis, were included in this study after written informed consent.

### Data registration

An open-source software package implemented in MATLAB was used for non-rigid registration, based on the demon algorithm [23, 24]. Gaussian smoothing with a sigma of 4 was applied to the velocity field each iteration for regularization. End-expiratory and end-inspiratory phases of the reconstructed 4D MRI data sets served as target image sets. All other reconstructed phases, with a total number of n, were registered onto the corresponding target image set using two different registration schemes, referred to as direct (dir-Reg) and next-neighbour (nn-Reg) registration. The different registration schemes are exemplarily illustrated in Fig. 1 with the end-expiratory phase as target and *n* = 5:

- nn-Reg (right): A chain of alternating registration and averaging steps being used. Phase 5 is first registered on the adjacent phase 4. The resulting phase 54 and phase 4 are then averaged before this data set is registered on phase 3 and averaged again. In the last registration step, data set 5432 is registered on the target and then averaged once more.
- dir-Reg (left): Each phase is directly registered on the target resulting in data sets 51, 41, 31, 21 and then averaged.

Both registration schemes lead to a data set, which preserves the anatomy of the target due to registration containing the signal intensity of the complete 4D data set due to averaging.

### Quantitative data evaluation

Regions of interest (ROIs) were drawn in the liver, lung and the heart respectively for calculation of the coefficient of variation (CV)

$$ CV=\frac{{}^{\sigma }R OI}{{}^{\mu }R OI}, $$

as an image quality measurement [17] with the standard deviation σ_ROI_ and the mean μ_ROI_ of the ROIs. The CV describes local image homogeneity. It was used to compare the registration schemes regarding artifacts introduced by registration. The mean CV of ROIs, drawn in three adjacent slices for each organ, was used for evaluation.

**Fig. 2:**
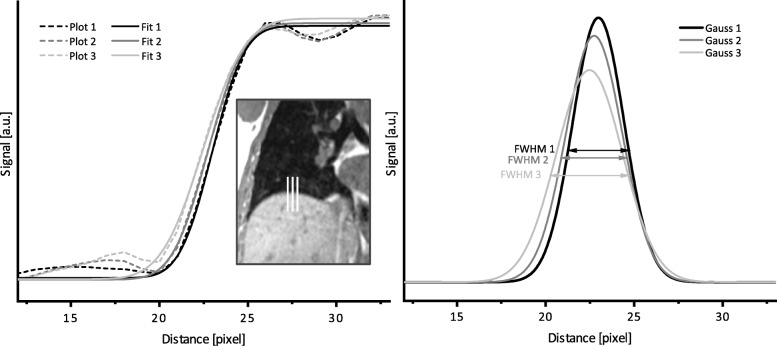
Schematic overview of sharpness evaluation. Left: Exemplary line plots over the diaphragm and corresponding error function fit. Right: Corresponding Gaussian curves and calculated FWHM used as sharpness measure

Image sharpness was calculated using 10 manually defined intensity plots across edges at the lung-liver interface and tumor boundaries. Each profile is individually fitted with an error function [25]. The Full Width at Half Maximum (FWHM) of the underlying Gaussian function is then calculated and used as a measure of image sharpness expressed in pixel. A small FWHM means high sharpness. Figure 2 exemplarily shows three edge profile plots and the error function fits on the left. The calculated FWHM with corresponding Gaussian functions are displayed on the right. CV and sharpness were calculated for end-expiratory and end-inspiratory phase of the target and the registered data sets dir-Reg and nn-Reg for volunteer and patient measurements. CV values were normalized to the corresponding target sets.

Wilcoxon matched-pair signed-rank test was used for comparison between the three data sets. Statistical significance was considered for *p* <  0.05. Additionally, the total reconstruction and registration times were assessed for all patient measurements for evaluation of clinical suitability.

### Qualitative data evaluation

Image scoring of the patient measurements was performed independently by two radiation oncologists and one radiologist all having at least 5 years of experience in reading MR images. Readers were blinded to clinical information and applied reconstruction algorithm. Corresponding target, dir-Reg, and nn-Reg data sets were presented simultaneously to the reader for each patient and respiratory phase (end-expiration and end-inspiration). The order of the three data sets, patients and respiratory phase was randomly changed. Criteria for scoring were: (1) noise level (e.g. in the liver or the lung), (2) sharpness (identification and delineation of tumors, the sharpness of the diaphragm and small liver and lung vessels) and (3) overall image quality. A four-point Likert scale was used for scoring: Score of 1 poor; score of 2 fair; score of 3 good, score of 4 very good.

## Results

4D MRI data sets could be successfully reconstructed for all volunteers and patients. Average 4D MRI reconstruction times of the patient measurements were about 330 min and registration times (including inspiration and expiration) 157 min for nn-Reg and 361 min for dir-Reg.

**Fig. 3:**
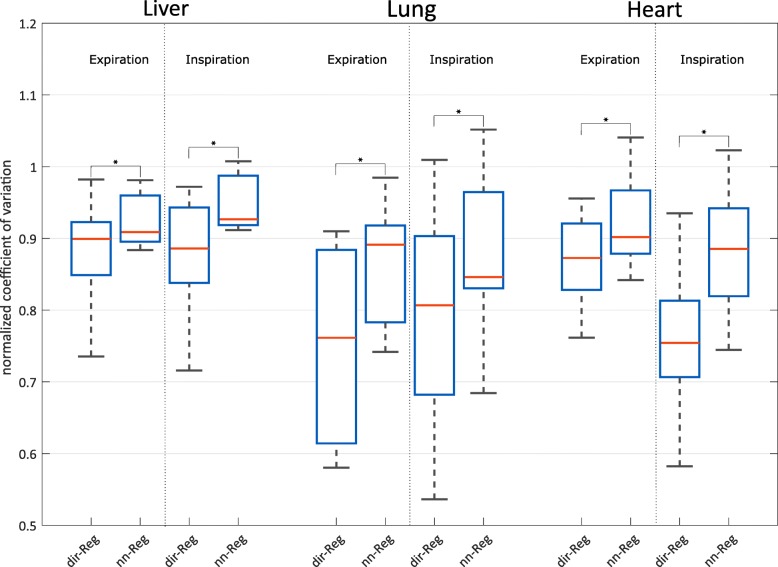
Boxplots of the normalized CV for the two registration schemes for expiration and inspiration of different body regions liver, lung, and heart. Statistical significant differences (*p* < 0.05) in CVbetween the schemes are marked with stars

Figure 3 shows the boxplots of the normalized CV for the different registration schemes. Volunteer and patient data sets were used for evaluation. The median CV of all single organs of the two registration schemes decreased compared to the target for expiration and inspiration. The median CVs of the dir-Reg scheme were lower compared to nn-Reg and statistically significant differences (*p* <  0.05) between the CVs of the two schemes (marked as stars) were found in all cases. The average CV over all organs was 5.6 ± 5.3% lower for expiration and 7.0 ± 4.2% lower for inspiration comparing dir-Reg with nn-Reg.

**Fig. 4:**
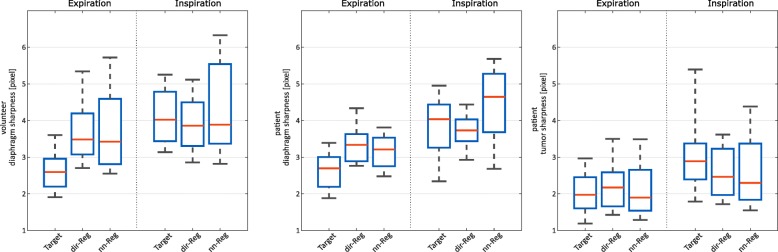
Boxplots showing the sharpness of volunteer diaphragm (left), patient diaphragm (middle), and tumor (right) in expiration and inspiration for the registration schemes and the target

**Fig. 5:**
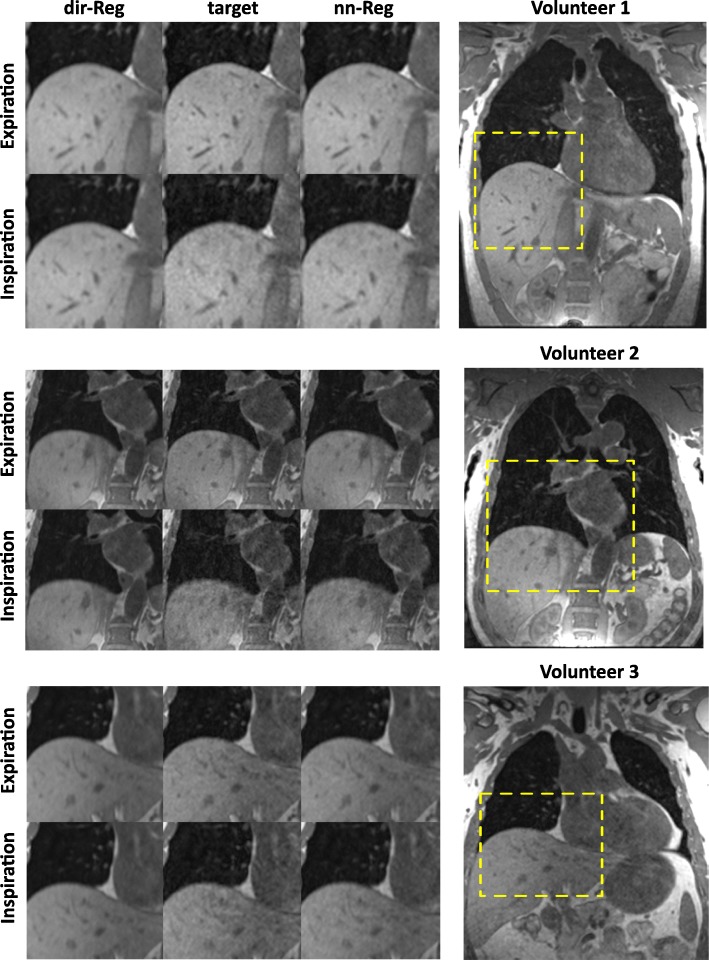
Comparison between exemplarily coronal slices of the registered and target image sets of three volunteers. Expiration and inspiration images are displayed. Zoomed regions (depicted in the right images) are shown to emphasize differences in sharpness and signal intensity

The results of the sharpness measurements are displayed in Fig. 4. Median expiration diaphragm and tumor sharpness are higher (smaller FWHM) compared with corresponding inspiration sharpness. Target diaphragm sharpness is the highest in case of expiration whereas dir-Reg sharpness is the highest in case of inspiration. Tumor sharpness is higher in all cases compared to diaphragm sharpness. The nn-Reg registration shows the highest interquartile ranges in all comparisons. Table 1 shows the results of the Wilcoxon signed-rank test used for comparison of sharpness between the data sets. Insignificant differences could be found in different comparisons and breathing states. Figure 5 shows a qualitative comparison between coronal slices of the registered and target image sets of three volunteers. Zoomed regions (depicted in the right images) are shown to emphasize differences in sharpness and signal intensity.

**Table 1:** *P*-values of the statistical comparison of the sharpness between volunteer and patient data sets in expiration and inspiration

	Comparison	Volunteer Expiration	Volunteer Inspiration	Patient Diaphragm Expiration	Patient Diaphragm Inspiration	Patient Tumor Expiration	Patient Tumor Inspiration
*p*-Value	Target – dir	< 0.01	0.04	< 0.01	0.19	0.02	0.01
	Target – nn	< 0.01	0.31	< 0.01	< 0.01	< 0.01	< 0.01
	Dir - nn	0.29	0.01	< 0.01	< 0.01	0.05	0.9

**Fig. 6:**
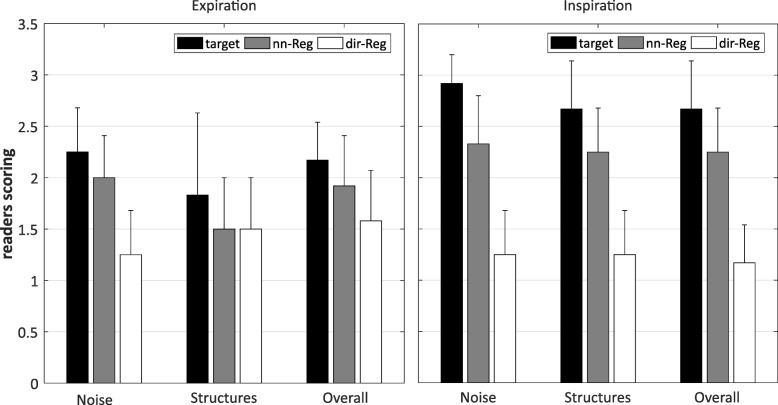
Mean and standard deviation of the readers scoring derived from the patient measurements. Target and registration schemes scores of the single categories are displayed for expiration and inspiration

**Fig. 7:**
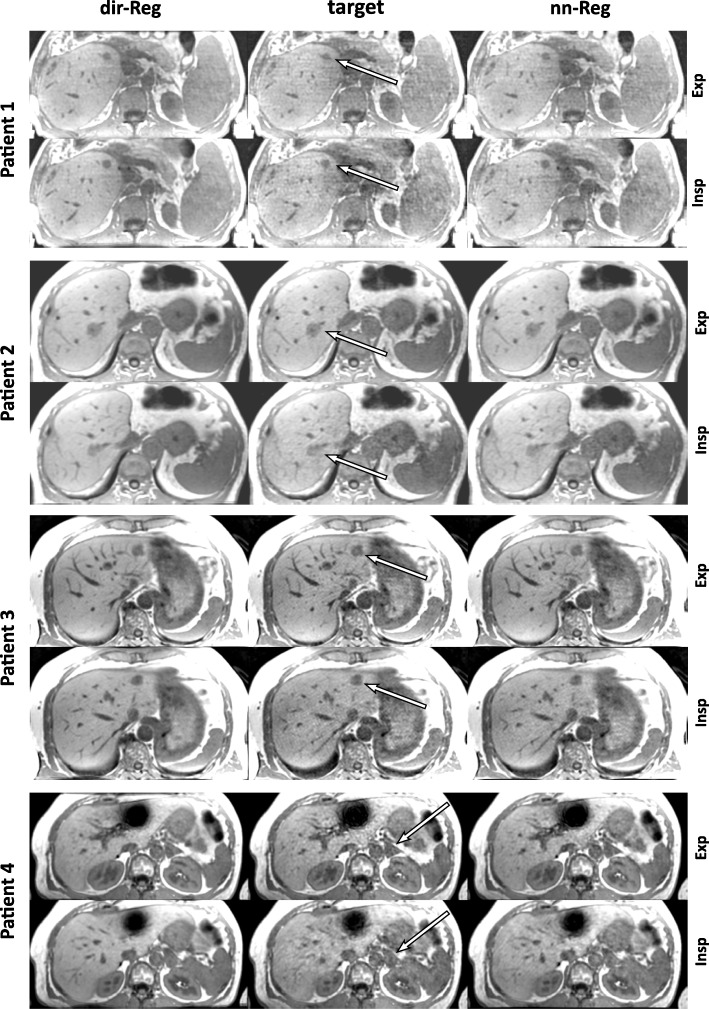
Exemplary transversal slices of the registered and target image sets for each patient containing a lesion (arrows). Expiration and inspiration images are displayed for comparison. The arrows illustrate the inferior image quality of the target image sets

Mean scores of the blinded reading of the patient data sets are displayed in Fig. 6. Expiration and inspiration target scores are lower in all criteria compared with both registration schemes. Noise and overall image quality are rated higher for dir-Reg than nn-Reg. The structure delineation of the registration schemes was rated equally. Inspiration dir-Reg scores are higher in all categories compared to nn-Reg. Corresponding expiration rating is higher than inspiration for the target and nn-Reg whereas dir-Reg structure and overall image quality scores are higher and noise the same. Over all categories, mean expiration scores were 2.92 ± 0.18 for the target, 3.19 ± 0.22 for nn-Reg and 3.56 ± 0.14 for dir-Reg and mean inspiration scores 2.25 ± 0.12 for the target, 2.72 ± 0.04 for nn-Reg and 3.78 ± 0.04 for dir-Reg. The patient images in Fig. 7 exemplarily visualize these findings. The artifacts in the lower patient images were caused by metal clips. Dir-Reg images show a more homogeneous signal compared with nn-Reg and especially with the target. In the case of inspiration, the image quality of the target and nn-Reg images strongly decreases compared to dir-Reg images.

## Discussion

Two different non-rigid image registration schemes are used to increase the signal intensity of end-expiratory and end-inspiratory phases while preserving the motion artifact reduction. To this end, all phases are registered onto either end-expiration or end-inspiration serving as targets. The results are compared amongst the schemes and the corresponding targets in terms of quantitative evaluation of image homogeneity (coefficient of variation) and sharpness as well as qualitative expert reader rating. The CV was used for evaluation because SNR measurements using signal and noise regions are not reliable if multichannel and parallel imaging reconstructions are used [17, 26].

Both registration schemes used in this work lead to lower median CV values (higher homogeneity) compared to the targets (Fig. 3). Median CV values were lower for dir-Reg compared to nn-Reg. This can be explained by the fact that the single phases in case of nn-Reg are contributing not equally, leading to less efficient suppression of noise due to averaging. Boldea et al. [27] used similar registration schemes with 4D-CT data to calculate patient-specific tumor motion and hysteresis. Accuracy and consistency were globally evaluated and the differences between the schemes were found to be statistically not significant. However, they state that differences may occur in a local evaluation due to various sources of errors [28] having a different impact on the two registration schemes. In contrast, the local evaluation of CV in this study showed significant differences between the registration schemes.

The image quality of inspiration is usually lower compared to expiration using equal bin sizes because resting times in end-inspiratory phases are shorter and the position is not as reproducible compared to end-expiratory phases (see respiration curve in Fig. 1). This leads to increased motion artifacts, noise and under-sampling artifacts due to less accepted data within the gating process [29, 30]. As a result, random intestinal peristaltic and heart motion which cannot be gated are also more pronounced due to the reduced averaging effect [31, 32]. These factors influence sharpness and CV evaluation as illustrated in Figs. 1, 4 and 5. Median inspiration sharpness is lower in all comparisons than the corresponding expiration sharpness (Fig. 4). Moreover, median target sharpness can even be improved by registration in case of inspiration. The sharpness of structures strongly depends on the proximity to the diaphragm. Consequently, tumor sharpness is much higher compared to diaphragm sharpness in this study because most of the tumors are located further away. Statistically significant differences could be found in most of the comparisons (Table 1).

The qualitative rating results of the patient measurements confirm the findings discussed above (Fig. 6). Expiration target images are rated better in all categories compared to inspiration. Targets were rated lower than both registration schemes. The dir-Reg scheme got the best noise and overall image quality scores. Structure scores of dir-Reg and nn-Reg were almost the same for expiration but dir-Reg scored higher in inspiration. Dir-Reg inspiration scores are even better than the corresponding expiration scores. This may be explained by the simultaneous reading used in this work. The poor image quality of the inspiration target image could influence the perception of the benefit in image quality.

Buerger et al. used a golden-radial phase encoding acquisition with respiratory self-gating to reconstruct a high-quality reference image and various higher under-sampled phase-resolved images [33]. Non-rigid registrations are subsequently applied between the reference and all other under-sampled phases, leading to high-quality 4D images. In contrast to this work, our focus was to improve the image quality of end-expiration and end-inspiration phases. All the acquired data is used for image quality enhancement of these two phases respectively. However, the proposed method by Buerger et al. could additionally be applied to exploit the same acquired data.

Four-dimensional imaging is essential in target volume and organ at risk delineation in radiotherapy treatment planning for moving targets. The total extent of tumor motion between expiration and inspiration is required in some treatment planning concepts [1, 2]. The proposed method could be used to assist in the delineation process in this context. However, average 4D MRI reconstruction and registration times are too long because MRI data sets should be available on the same day as 4D CT to assist in the delineation process. 4D MRI reconstruction and registration times are varying due to individual breathing leading to various under-sampling patterns responsible for parallel imaging performance, initial image quality and different volume sizes used in the registration process.

Almost the whole volume in cranio-caudal and anterior-posterior direction was used for quantitative and qualitative evaluation in this work. Focusing on the lesions will decrease the volume and hence registration time. The increasing use of image registration in different applications in the context of MR guided radiotherapy [33–36], registration algorithms capable of parallel computing [37] and the use of different parallel imaging reconstructions will lead to further time reductions [38]. The physician should decide if registration in case of expiration is necessary or not and if nn-Reg should be preferred because of shorter reconstruction times if image quality is supposed to be not significantly lower compared to dir-Reg Another requirement would be the same patient positioning in the CT and the MR to avoid inaccuracies in image fusion. Therefore, a wide-bore scanner would be preferable because it would allow for a comparison to 4D-CT for more patients. However, the method described in this paper does not depend on the scanner type though because the acquisition sequence can be implemented on every MRI scanner. Additionally, gradient nonlinearity has definitely to be corrected for reliable and accurate delineation.

## Conclusion

In this work, end-expiratory and inspiratory phases of a retrospective respiratory self-gated 4D MRI data set of volunteers and patients were used as targets for non-rigid image registration. All other phases of the 4D MRI were registered onto these targets using two different registration schemes. It was shown that image quality of the target images can be significantly increased while motion artifact reduction is preserved allowing for improved lesion detection.

## Data Availability

The datasets used and/or analysed during the current study are available from the corresponding author on reasonable request.

## Abbreviations

OAR: Organ at risk
RT: Radiotherapy treatment
CT: Computed tomography
MRI: Magnetic resonance imaging
CV: Coefficient of variation
SNR: Signal to noise ratio
ROI: Region of interest
FWHM: Full with at half maximum
